# The expression level of *HJURP *has an independent prognostic impact and predicts the sensitivity to radiotherapy in breast cancer

**DOI:** 10.1186/bcr2487

**Published:** 2010-03-08

**Authors:** Zhi Hu, Ge Huang, Anguraj Sadanandam, Shenda Gu, Marc E Lenburg, Melody Pai, Nora Bayani, Eleanor A Blakely, Joe W Gray, Jian-Hua Mao

**Affiliations:** 1 Life Sciences Division, Lawrence Berkeley National Laboratory, One Cyclotron Road, Berkeley, CA 94720, USA; 2 Department of Laboratory Medicine, UCSF Helen Diller Family Comprehensive Cancer Center, University of California, 1600 Divisadero Street, San Francisco, CA 94143, USA; 3 Department of Pathology and Laboratory Medicine, Boston University School of Medicine, 715 Albany Street, Boston, MA 02118, USA; 4 Department of Molecular and Cell Biology, University of California, 142 LSA #3200, Berkeley, CA 94720, USA

## Abstract

**Introduction:**

HJURP (Holliday Junction Recognition Protein) is a newly discovered gene reported to function at centromeres and to interact with CENPA. However its role in tumor development remains largely unknown. The goal of this study was to investigate the clinical significance of *HJURP *in breast cancer and its correlation with radiotherapeutic outcome.

**Methods:**

We measured *HJURP *expression level in human breast cancer cell lines and primary breast cancers by Western blot and/or by Affymetrix Microarray; and determined its associations with clinical variables using standard statistical methods. Validation was performed with the use of published microarray data. We assessed cell growth and apoptosis of breast cancer cells after radiation using high-content image analysis.

**Results:**

*HJURP *was expressed at higher level in breast cancer than in normal breast tissue. *HJURP *mRNA levels were significantly associated with estrogen receptor (ER), progesterone receptor (PR), Scarff-Bloom-Richardson (SBR) grade, age and Ki67 proliferation indices, but not with pathologic stage, ERBB2, tumor size, or lymph node status. Higher *HJURP *mRNA levels significantly decreased disease-free and overall survival. *HJURP *mRNA levels predicted the prognosis better than Ki67 proliferation indices. In a multivariate Cox proportional-hazard regression, including clinical variables as covariates, *HJURP *mRNA levels remained an independent prognostic factor for disease-free and overall survival. In addition *HJURP *mRNA levels were an independent prognostic factor over molecular subtypes (normal like, luminal, Erbb2 and basal). Poor clinical outcomes among patients with high *HJURP *expression were validated in five additional breast cancer cohorts. Furthermore, the patients with high *HJURP *levels were much more sensitive to radiotherapy. *In vitro *studies in breast cancer cell lines showed that cells with high *HJURP *levels were more sensitive to radiation treatment and had a higher rate of apoptosis than those with low levels. Knock down of *HJURP *in human breast cancer cells using shRNA reduced the sensitivity to radiation treatment. *HJURP *mRNA levels were significantly correlated with CENPA mRNA levels.

**Conclusions:**

*HJURP *mRNA level is a prognostic factor for disease-free and overall survival in patients with breast cancer and is a predictive biomarker for sensitivity to radiotherapy.

## Introduction

The centromere has long been recognized as a locus important for proper cell division and accurate partitioning of chromosomes into daughter cells [1-3]. Centromeres are the chromatin regions associated with kinetochores, which are massive multi-protein complexes that mediate chromosome segregation and the mitotic checkpoint [4]. There is mounting evidence that kinetochores become functionally unstable during oncogenesis resulting in segregation defects, chromosome instability, and cancer development [4-6].

Holliday Junction Recognition Protein (HJURP, also known as hFLEG1), which is a newly discovered gene, was reported to be overexpressed in lung cancer cells through genome-wide expression profile analysis [7]. By quantitative RT-PCR, Valente et al found that the *HJURP *expression levels significantly differ between glioblastoma resection tumor and non-neoplastic white matter [8]. Additionally it was observed that the expression level of *HJURP *in glioblastoma was changed about nine fold compared to typically benign pilocytic astrocytomas by microarray profile analysis [9]. It has also been reported that *HJURP *is involved in DNA double-strand break repair pathway through interaction with *MSH5 *and *NBS1 *[7]. Recently two groups have shown that *HJURP *functions at the level of the centromere, and is required for centromere protein A (CENPA) centromeric localization, for loading of new CENPA nucleosomes, and for accurate chromosomal segregation [10-12]. A majority of cancer cells tend to gain and lose chromosomes at each mitotic division and are found to be aneuploid and chromosomally instable. Thus these findings support the hypothesis that alterations in *HJURP *might play an important role in cancer development. We investigated whether altered expression levels of *HJURP *are associated with adverse clinical outcomes using cohorts of patients with breast cancer.

## Materials and methods

### Cell lines and cell lysates

The names of cell lines used in our investigations are listed in Table 1. The derivation, sources, and maintenance of most of the breast cancer cell lines used in this study have been reported previously [13] or were provided in Table 2. These cell lines have been previously analyzed for genomic aberrations by comparative genomic hybridization (CGH) and for gene-expression profiles using Affymetrix microarrays (Santa Clara, CA, USA) [13]. The information on growth conditions of additional cell lines was listed in Table 2. Cells at 50% to 75% confluence were washed in ice-cold phosphate buffered saline (PBS). Then cells were extracted with a lysis buffer (containing 50 mM HEPES (pH 7.5), 150 mM NaCl, 25 mM β-glycerophosphate, 25 mM NaF, 5 mM EGTA, 1 mM EDTA, 15 mM pyrophosphate, 2 mM sodium orthovanadate, 10 mM sodium molybdate, 1% Nonidet-P40, 10 mg/ml leupeptin, 10 mg/ml aprotinin, and 1 mM PMSF). Cell lysates were then clarified by centrifugation and frozen at -80°C. Protein concentrations were determined using the Bio-Rad BCA protein assay kit (Cat# 23227, Pierce Biotechnology, Rockford, IL, USA).

**Table 1:** The list of breast cancer cell lines and immortalized non-malignant mammary epithelial cells used in these investigations.

Set 1		Set 2		Set 3		Set 4		Set 5		Set 6		Set 7	
						
Lane	Name	Lane	Name	Lane	Name	Lane	Name	Lane	Name	Lane	Name	Lane	Name
1	SKBR3	1	SKBR3	1	SKBR3	1	SKBR3	1	SKBR3	1	SKBR3	1	SKBR3
2	MCF12A	2	MCF12A	2	MCF12A	2	MCF12A	2	MCF12A	2	MCF12A	2	MCF12A
3	600MPE	12	MDAMB134	21	BT483	30	184A1N4	39	DU4475	48	HCC1395	57	MX-1
4	AU565	13	MDAMB157	22	HCC70	31	184B5	40	SUM1315M02	49	HCC1428	58	SUM102
5	BT20	14	MDAMB175	23	HCC1187	32	HCC38	41	HCC1954	50	HCC1806	59	SUM190
6	BT474	15	MDAMB231	24	HCC1500	33	HCC202	42	SUM44PE	51	HCC1937	60	HCC1419
7	BT549	16	MDAMB361	25	MCF10A	34	HCC1143	43	SUM52PE	52	HCC2185	61	HCC3153
8	CAMA1	17	MDAMB415	26	MDAMB453	35	HCC1569	44	SUM149PT	53	HCC2218	62	S1
9	HBL100	18	MDAMB435	27	ZR751	36	HCC1599	45	SUM159PT	54	HCC1599	63	T4
10	Hs578T	19	T47D	28	ZR7530	37	LY2	46	SUM185PE	55	UACC893	64	MDAMB231-Gray
11	MCF7	20	UACC812	29	ZR75B	38	SUM225	47	SUM225CWN	56	SUM229	65	MDAMB231-ATCC

**Table 2:** Additional cell line growth conditions and subtypes

Lane	Name	Subtype*	Medium	Culture condition
30	184A1N4	N	MEGM^a^	37°C, 5% CO_2_
31	184B5	N	MEGM^a^	37°C, 5% CO_2_
36	HCC1599	Basal A	RPMI1640+10%FBS^b^	37°C, 5% CO_2_
48	HCC1395	Basal B	RPMI1640+10%FBS	37°C, 5% CO_2_
50	HCC1806	Basal A	RPMI1640+10%FBS	37°C, 5% CO_2_
53	HCC2218	Luminal	RPMI1640+10%FBS	37°C, 5% CO_2_
54	HCC1599	Basal A	RPMI1640+10%FBS	37°C, 5% CO_2_
55	UACC893	Luminal	DMEM+10% FBS	37°C, 5% CO_2_
56	SUM229PE	N/A	Ham's F12+5% FBS+IH^c^	37°C, 5% CO_2_
57	MX-1	N/A	RPMI1640+10%FBS	37°C, 5% CO_2_
58	SUM102PT	Basal A	Ham's F12+IHE^d^	37°C, 5% CO_2_
60	HCC1419	Luminal	RPMI1640+10%FBS	37°C, 5% CO_2_
62	S1	N	H14 medium +10 ng/ml EGF	37°C, 5% CO_2_
63	T4	Basal B	H14 medium^e^	37°C, 5% CO_2_
64	MDAMB231-Gray	Basal B	DMEM+10% FBS	37°C, 5% CO_2_
65	MDAMB231-ATCC	Basal B	DMEM+10% FBS	37°C, 5% CO_2_

^a^: Clonetics MEBM (no Bi Carbonate)+Insulin(5 ug/ml)+Transferrin(5 ug/ml)+Hydrocortisone(0.5 ug/ml) +EGF(5 ng/ml) +Isoprorternol

^b^: Fetal bovine serum (FBS)

^c^: Ham's F12 + 5% FBS + IH {insulin (5 ug/ml) + Hydrocortisone (1 ug/ml) + HEPES (10 mM)}

^d^: Ham's F12 + IHE {insulin (5 ug/ml) + HEPES (10 mM) + EGF (10 ng/ml)}

^e^: H14 medium: DMEM/F12 (GIBCO/BRL) with 250 ng/ml insulin, 10 μg/ml transferrin, 2.6 ng/ml sodium selenite, 10^-10 ^M estradiol, 1.4 × 10^-6 ^M

*: N: non-malignant(immortalized), N/A: no data

### Western blot

For Western blots, 10 μg of protein extracts per lane were electrophoresed with denaturing sodium doedecyl sulfate (SDS)-polyacrylamide gels (4% to 12%), transferred to PVDF membranes (Millipore, Temecula, CA, USA), and incubated with HJURP antibody 1:500 (Rabbit, HPA008436, Sigma-Aldrich, St. Louis, MO, USA) and actin (goat, sc-1616, Santa Cruz Biotechnology, Santa Cruz, CA, USA) diluted with blocking buffer (927-40000, LI-COR Biosciences, Lincoln, NE, USA) The membranes were washed four times with TBST and treated with 1:10,000 dilution of Alex Fluor 680 donkey anti-rabbit (A10043, Invitrogen, Carlsbad, CA, USA) and IRDye 800CW conjugated donkey anti-goat (611-731-127, Rockland, Gilbertsville, PA, USA) to detect *HJURP *and actin respectively. The signals were detected by infrared imaging (LI-COR Biosciences, Lincoln, NE, USA). Images were recorded as TIFF files for quantification.

### Protein quantification

Protein levels were measured by quantifying infrared imaging recorded from labeled antibodies using Scion Image [14]. For each protein, the blots were made for 7 sets of 11 cell lines, each set including the same pair (SKBR3 and MCF12A) to permit intensity normalization across sets. A basic multiplicative normalization was carried out by fitting a linear mixed effects model to log intensity values, and adjusting within each set to equalize the log intensities of the pair of reference cell lines across the sets.

### Tumor samples

Detailed patient information has been described in our previous studies [15]. This analysis is based on previously reported comparative genomic hybridization (CGH) and a gene expression profile of 130 tumors from UC San Francisco and the California Pacific Medical Center collected between 1989 and 1997.

### Validation

The association of *HJURP *expression levels and survival among patients with breast tumors was examined in existing microarray data sets of primary tumor samples that had been profiled with an Affymetrix microarray assay (either HG-U133A or HG U133 Plus 2.0) ((GEO:GSE1456), (GEO:GSE7390), (GEO:GSE2034), (GEO:GSE4922)) or Agilent oligo microarray (Santa Clara, CA, USA)(Table 3). Probe 218726_at and 20366 (GenBank: NM_018410) were used to measure *HJURP *expression in Affymetrix and Agilent GeneChip, respectively. The process data from GEO website were downloaded for analysis.

**Table 3:** Information of gene expression datasets used in this study

Dataset	GEO access number or web location	Radiotherapy	Reference
1	GSE1456	Not available	[21]
2	GSE7390	Not available	[22]
3	NKI [26]	82.4% patients	[23]
4	GSE2034	86.7% patients	[24]
5	GSE4922	Not available	[25]

### *HJURP *shRNA construct

The shRNA sequences were (forward) 5'-GATCCCC GAGCGATTCATCTTCATCA TTCAAGAGA TGATGAAGATGAATCGCTC TTTTTGGAAA-3' and (reverse) 5'-AGCT TTTCCAAAAA GAGCGATTCATCTTCATCA TCTCTTGAA TGATGAAGATGAATCGCTC GGG-3' synthesized from IDT (Integrated DNA Technologies, Inc., San Diego, CA, USA). *HJURP *shRNA was cloned into BglII and HindIII cleavage sites of pSUPER.retro.puro vector based on manufactory's instruction (OligoEngine, Seattle, WA, USA). *HJURP *shRNA expression vector were confirmed by direct DNA sequencing.

### Retroviral packaging and infection

*HJURP *shRNA (or empty) retroviral vectors along with packaging system pHit60 and pVSVG vectors were then co-transfected into the HEK 293 Phoenix ampho packaging cells (ATCC, Manassas, VA, USA) by using FuGENE6 transfection reagent (Roche, Lewes, UK) according to the instruction to produce retroviral supernatants. Forty-eight hours after transfection, the virus-containing supernatant was filtered through a 0.45 μm syringe filter. Retroviral infection was performed by adding filtered supernatant to a MDAMB231 cell line cultured on 10 cm dishes with 50% confluent in the presence 4 ug/ml of polybrene (Sigma, St. Louis, MO, USA). Six hours after infection, the medium was changed with fresh medium. After 48 hours, infected cells were selected by adding 5 μg/ml puromycin (Sigma) to the culture medium for 72 hours and then maintained in complete medium with 2 μg/ml puromycin. Down-regulation of *HJURP *expression was confirmed by Western blot analysis.

### High content imaging to assess cell number and apoptotic cells

The effects on cell growth and apoptosis were assessed by a Cellomics high-content image screening system (Cellomics, Thermo Fisher Scientfic Inc., Pittsburgh, PA, USA) after breast cancer cells exposed to a single dose of 0 (sham), 1, 2, 4, 6, 8 or 10 Gy X-ray radiation emitted from an irradiator (model 43855F, Faxitron X-ray Corporation, Lincolnshire, IL, USA). Live cells in 96 well plates with six replicates from each treatment were stained with 1 μmol/L YO-PRO-1 positive cells.

### Statistical analysis

Spearman's correlation coefficient and test were used to examine the relationship between *HJURP *mRNA level and its protein level in the cell line studies, and the relationship with age, tumor size in the tumor studies, and CENPA mRNA level. The association between *HJURP *mRNA level and clinical factors, such as estrogen receptor (ER), progesterone receptor (PR), ERBB2 and lymph node status, pathological stage, Scarff-Bloom-Richardson (SBR) grade, was analyzed by Mann-Whitney U (for two groups) or Kruskal-Wallis H (for more than two groups) test. Kaplan-Meier plots were constructed and a long-rank test was used to determine differences among disease free and overall survival curves according to *HJURP *expression level or radiotherapy. Multivariate analyses were carried out to examine whether *HJURP *expression is an independent prognostic factor for survival when adjusting for other covariates (age, ER, PR, lymph node, pathologic stage, SBR grade, tumor size) or the molecular subtypes (normal like, luminal, Erbb2 and Basal) using Cox proportional-hazard regression. In addition, the relation between *HJURP *expression and survival was explored in microarray data sets by dividing the cases from each cohort into a group with high (top one-third), moderate (middle one-third), and low (bottom one-third) level of expression. All analyses were performed by SPSS 11.5.0 for Windows. A two-tailed *P*-value of less than 0.05 was considered to indicate statistical significance.

## Results

### *HJURP *is overexpressed in breast cancer

**Figure 1:**
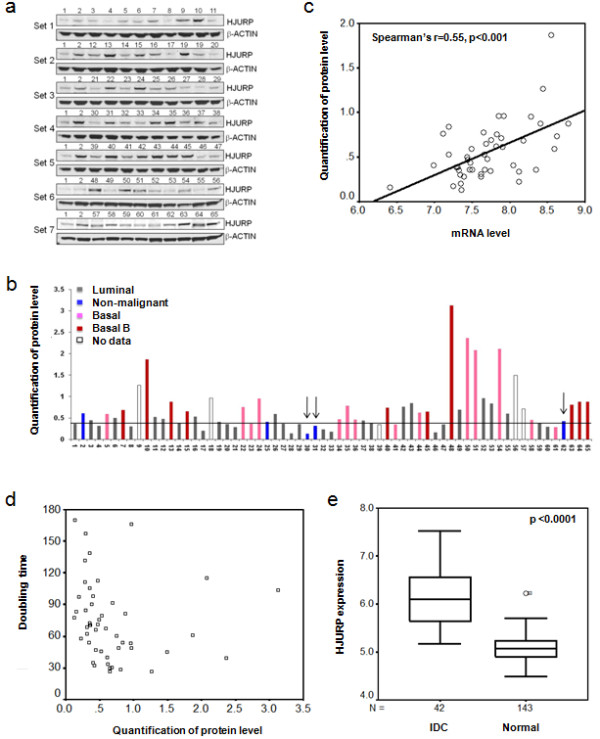
***HJURP *is overexpressed in human breast cancer cell lines and primary breast tumors**. **(a) **Protein levels of *HJURP *(Holliday junction recognition protein) in a large panel of human breast cancer cell lines and immortalized non-malignant mammary epithelial cells were assessed by Western blotting. Samples 30, 31 and 62 are immortalized non-malignant mammary epithelial cells 184A1N4, 184B5 and S1 respectively. **(b) **Normalized quantification of HJURP protein levels in the cell lines using Scion Image software are shown. The arrows indicate the immortalized non-malignant mammary epithelial cells 184A1N4, 184B5, and S1 respectively. The line shows M+1.95*SE where M is mean of 184A1N4, 184B5 and S1 protein levels and SE is standard error of 184A1N4, 184B5 and S1 protein levels. Protein level above this line was defined as overexpression. About 50% breast cancer cell lines have overexpression of *HJURP*. **(c) **Figure 1c shows the correlation between mRNA and protein levels of *HJURP *in human breast cancer cell lines. *HJURP *expression is measured as log_2 _(probe intensities) by Affymetrix microarray. The detail for protein quantification refers to Materials and Methods. R was Spearman's rho correlation coefficient. The two-tailed *P *-value was obtained from Spearman correlation test. **(d) **The *HJURP *protein level has a negative and significant correlation with the doubling times of cell lines. **(e) ***HJURP *mRNA expression level is significantly evaluated in invasive ductal carcinomas (IDC) in comparison to normal breast ducts. *HJURP *mRNA expression is assessed by Affymetrix microarray. *HJURP *expression is measured as log_2 _(probe intensities). The microarray data were found in Gene Expression Omnibus (GEO) database GEO accession numbers [GEO:GSE10780] [16].

We examined the protein levels of *HJURP *in a large panel of human breast cancer cell lines and immortalized non-malignant mammary epithelial cells, which have been analyzed for genomic aberrations by comparative genomic hybridization (CGH) and for gene-expression profiles using Affymetrix microarrays [13]. Although we found few genetic alterations in the *HJURP *locus by inspection of these CGH microarray data, the protein levels of *HJURP *were elevated in about 50% of these breast cancer cell lines when compared to immortalized but non-malignant mammary epithelial cells 184A1N4, 184B5, and S1 (Figure 1a, b). In order to determine whether mRNA expression reflected protein levels, we quantified and normalized *HJURP *protein expression in each cell line and demonstrated a significant correlation between mRNA expression and protein levels (the Affymetrix probe for *HJURP *is 218726_at: Spearman's correlation coefficient R = 0.55, *P *< 0.001; Figure 1c). Next we examined whether *HJURP *protein level is associated with cell proliferation. In order to do so, we measured the doubling time for each cell line and found that the doubling time of cell lines was negatively correlated with *HJURP *protein levels (Spearman's correlation coefficient R = -0.395, *P *= 0.005; Figure 1d). Furthermore, *HJURP *mRNA levels in invasive ductal carcinomas (IDC) were statistically significantly higher than its levels in the normal breast ducts (*P *< 0.0001) (Figure 1e) [16].

### *HJURP *mRNA level is an independent prognostic biomarker for poor clinical outcome

**Figure 2:**
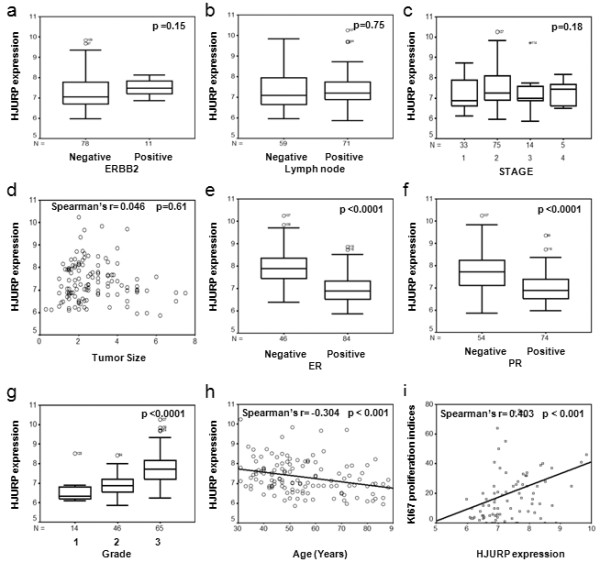
**Association of *HJURP *mRNA levels with clinic and pathological factors in patients with breast cancer**. There was no significant association between HJUPR mRNA levels and **(a) **ERBB2 (erythroblastic leukemia viral oncogene homolog 2) status, or **(b) **lymph node status, or **(c) **pathological stage or **(d) **tumor size. There were significant higher mRNA levels of *HJURP *in **(e) **estrogen receptor (ER) negative patients, **(f) **progesterone receptor (PR) negative patients; higher mRNA levels of HJURP were significantly associated with **(g) **high SBR grade, **(h) **younger age, and **(i) **Ki67 proliferation indices. *HJURP *expression is measured as log_2 _(probe intensities) by Affymetrix microarray. The two-tailed *P*-values were obtained by Mann-Whitney U test for ERBB2, lymph node, ER and PR status, Kruskal-Wallis H test for pathological stage and SBR grade, and Spearman correlation for size, age, and Ki67 proliferation indices.

**Figure 3:**
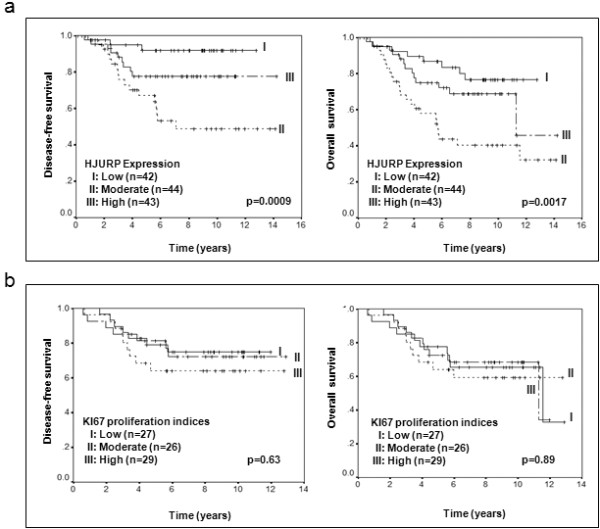
**The impact of *HJURP *expression and Ki67 proliferation indices on the disease-free and overall survival**. Figure 3 shows Kaplan-Meier survival curves for breast cancer patients according to tumor expression of *HJURP*. The patients from each cohort were divided into a group with high (top one-third), moderate (middle one-third) and low (bottom one-third) level of *HJURP *expression. *HJURP *expression is measured log_2 _(probe intensities) as in the microarray. The same criteria were used for Ki67 proliferation indices. *HJURP *mRNA expression was a significant prognostic factor for disease-free and overall survival, whereas Ki67 proliferation indices were not significantly associated with prognosis. **(a) **Kaplan-Meier survival curves for disease-free and overall survival are presented, while **(b) **shows the Kaplan-Meier survival curves for disease-free and overall survival based on Ki67 proliferation indices. The *P*-values shown were obtained from a long-rank test.

We assessed the association between *HJURP *mRNA levels and clinical factors and outcomes using a cohort of breast cancer patients in our previous studies [15]. *HJURP *expression level is measured as log_2 _(probe intensities) by Affymetrix microarray. In univariate analysis, *HJURP *mRNA levels were not associated with pathological stage, tumor size, ERBB2 positive, or lymph node positive status (Figure 2a, b, c, d). However, high *HJURP *mRNA levels were significantly associated with estrogen-receptor negative (ER-) (*P *< 0.0001), progesterone-receptor negative (PR-) *P *< 0.0001), advanced SBR grade (*P *< 0.0001), young age (*P *< 0.001) and Ki67 proliferation indices (*P *< 0.001) (Figure 2e, f, g, h, i). When we divided *HJURP *expression levels into three groups (low = bottom third, moderate = middle third, and high = top third), patients whose tumor with high *HJURP *expression levels had significantly shorter disease free survival (*P *= 0.0009) and overall survival (*P *= 0.0017) period using a Kaplan-Meier log rank analysis (Figure 3a). Interestingly, although *HJURP *expression significantly correlated with Ki67 proliferation indices, Ki67 proliferation indices are not significantly associated with both disease-free and overall survival (Figure 3b).

In multivariate analyses (including age, pathological stage, SBR grade, ER status, PR status, lymph node status, tumor size, *HJURP *mRNA levels), lymph node positive and high pathological stage were associated with poor disease free survival, whereas lymph node positive, big tumor size, and age were associated with poor overall survival (Table 4). *HJURP *expression level is an indicator of a poor prognosis for disease-free survival (hazard ratio, 2.05; 95% CI, 1.18 to 3.58; *P *= 0.011), and for overall survival (hazard ratio, 1.83; 95% CI, 1.11 to 3.01; *P *= 0.018) (Table 4).

**Table 4:** Results of multivariate analysis of independent prognostic factors in patients with breast cancer using Cox regression

	Disease-Free survival		Overall survival	
		
Factor	Hazard ratio (95% CI)	*P *value	Hazard ratio (95% CI)	*P *value
HJURP expression^+^	2.05 (1.18 to 3.58)	0.011	1.83 (1.11 to 3.01)	0.018
Lymph node (positive)	3.76 (1.16 to 12.25)	0.028	2.72 (1.08 to 6.88)	0.035
High Stage	2.23 (1.08 to 4.59)	0.030	1.85 (0.94 to 3.63)	0.075
Tumor size	1.32 (0.97 to 1.79)	0.079	1.34 (1.02 to 1.77)	0.038
Age (year)	1.01 (0.99 to 1.05)	0.33	1.03 (1.004 to 1.053)	0.022
High SBR Grade	0.76 (0.33 to 1.75)	0.52	1.00 (0.50 to 2.00)	0.99
ER (positive)	0.63 (0.21 to 1.94)	0.42	0.86 (0.33 to 2.25)	0.75
PR (positive)	0.90 (0.33 to 2.50)	0.84	0.95 (0.40 to 2.26)	0.91

^+ ^*HJURP *expression is measured as log_2 _(probe intensities) by Affymetrix microarray

**Figure 4:**
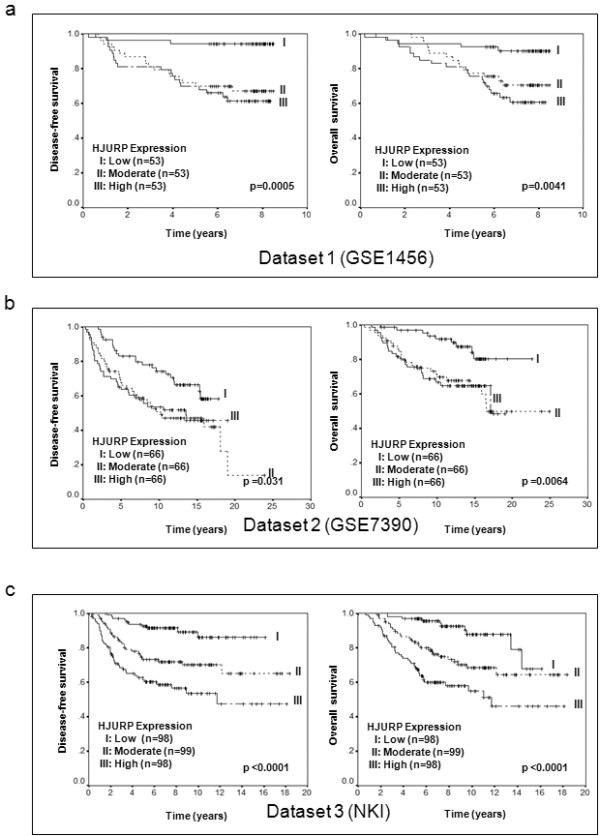
**Validation of the association between HUJRP mRNA and prognosis in three independent cohorts**. Kaplan-Meier survival curves for breast cancer patients according to tumor expression of *HJURP *are shown. The patients from each cohort were divided into a group with high (top one-third), moderate (middle one-third) and low (bottom one-third) level of *HJURP *expression. *HJURP *expression is measured log_2 _(probe intensities) as in the microarray. The significant association between *HJURP *mRNA and disease-free and overall survival was validated in three independent cohorts of patients with breast cancer. Parts **(a), (b) **and **(c) **show the Kaplan-Meier survival curves for disease-free and overall survival in Dataset 1 (GSE1456), Dataset 2 (GSE7390) and Dataset 3 (NKI) respectively. The *P*-values shown were obtained from a long-rank test.

**Figure 5:**
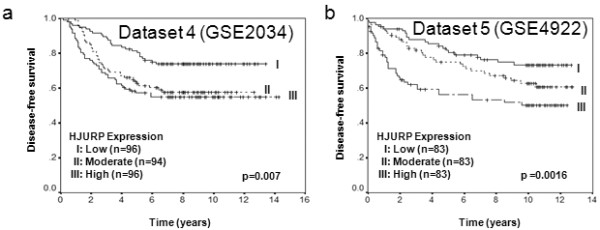
**Validation of the association between *HJURP *mRNA and disease-free survival in another two independent cohorts**. Kaplan-Meier survival curves for breast cancer patients according to tumor expression of *HJURP *are shown. The patients from each cohort were divided into a group with high (top one-third), moderate (middle one-third) and low (bottom one-third) level of *HJURP *expression. *HJURP *expression is measured log_2 _(probe intensities) as in the microarray. The significant association between *HJURP *mRNA and disease-free survival was further validated in two independent cohorts of patients with breast cancer. Parts **(a) **and **(b) **show the Kaplan-Meier survival curves for disease-free survival in Dataset 4 (GSE2034) and Dataset 5 (GSE4922). The *P*-values shown were obtained from a long-rank test.

To validate our findings, we used several independent breast cancer cohorts with previously reported microarray data deposited in the Gene Expression Omnibus (GEO) database [17], to compare mRNA level of *HJURP *in tumor tissue with patient survival (Table 3). In agreement with our initial findings, decreased disease-free and overall survival rate was associated with high mRNA level of *HJURP *in all of the datasets (Figures 4 and 5).

Finally, we investigated whether *HJURP *mRNA levels were an independent prognostic factor over molecular subtypes (normal like, luminal, Erbb2 and basal) using Cox regression. In order to do so, three data sets (reference 14, Dataset 1 and 3), in which the information of the molecular subtypes was available, were combined because there were few patients in each subtype using each data set. As showed in Table 5, both *HJURP *mRNA levels and molecular subtypes were independently significantly associated with survival.

**Table 5:** Both *HJURP *mRNA levels and molecular subtypes are independent prognostic factors in patients with breast cancer using Cox regression^#^

	Disease-Free survival		Overall survival	
		
Factor	Hazard ratio (95% CI)	*P *value	Hazard ratio (95% CI)	*P *value
**HJURP**		6.19E-07		0.00011
High vs Low	3.26 (2.01 to 5.28)	1.72E-06	3.23 (1.85 to 5.62)	3.65E-05
Moderate vs Low	3.34 (2.11 to 5.27)	2.3E-07	2.89 (1.68 to 4.95)	0.00012
**Molecular Subtypes**		0.0069		0.00012

^#^The results are obtained from the combination of three data sets (reference 14, Dataset 1 and 3) where the information of the molecular subtypes (Normal like, Luminal, Erbb2 and Basal) was available.

### *HJURP *mRNA level predicts the sensitivity to radiation treatment in breast cancer patients and cell lines

**Figure 6:**
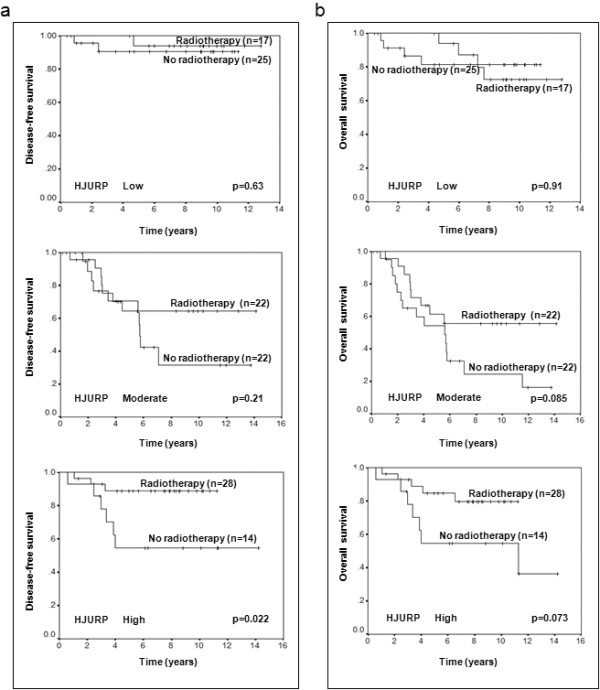
**The expression level of *HJURP *is a predictive factor for radiotherapy sensitivity**. Kaplan-Meier survival curves for breast cancer patients according to radiotherapy treatment are presented. Part **(a) **shows the survival curves for disease-free survival, while **(b) **shows survival curves for overall survival. The *P*-values shown were obtained from a long-rank test.

It has been reported that HJURP is involved in the DNA repair pathway, thus next we investigated whether the *HJURP *mRNA level is a predictive marker for radiotherapy in our cohort of breast cancer patients. As shown in Figure 6a, the radiotherapy significantly increased disease-free survival of patients within the high *HJURP *mRNA level group (*P *= 0.022) whereas radiotherapy did not within the low *HJURP *mRNA level group. The data showed a trend toward increased overall survival within the high and moderate *HJURP *mRNA level group (Figure 6b).

**Figure 7:**
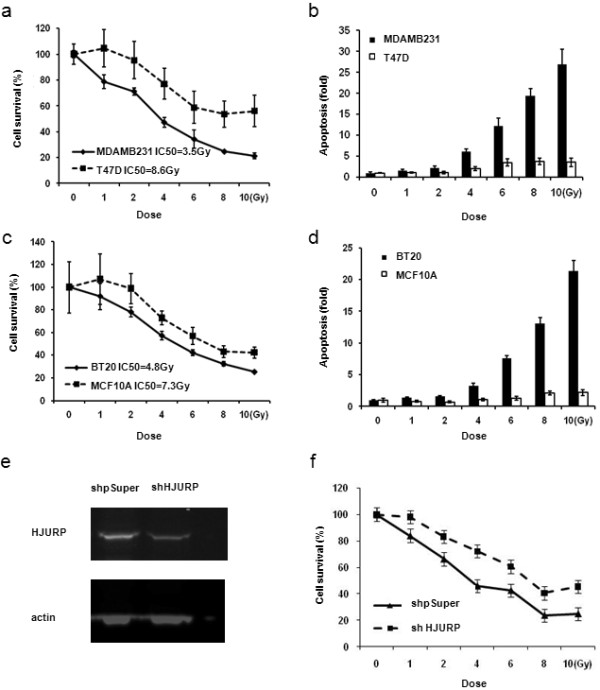
**The *HJURP *mRNA level in breast cancer cell lines predicts the sensitivity to radiation treatment**. Part **(a) **shows the percent of viable cells at 72 hours after different doses of radiation in breast cancer cell line MDAMB231 with a high level of *HJURP *and T47D with a low level of *HJURP*. MDAMD231 cells are more sensitive to radiation treatment than T47D cells. Figure 7**b **shows the fold change of apoptosis in comparison to control (no radiation) at 72 hours after the different dose of radiation in breast cancer cell line MDAMB231 and T47D. There are more apoptosis in MDAMB231 cells than T47D cells. Part **(c) **shows the percent of viable cells at 72 hours after the different dose of radiation in breast cancer cell line BT20 with high level of *HJURP *and MCF10A with low level of *HJURP*. BT20 cells are more sensitive to radiation treatment than MCF10A cells. Part **(d) **shows the fold change of apoptosis in comparison to control (no radiation) at 72 hours after the different dose of radiation in breast cancer cell line BT20 and MCF10A. There are more apoptosis in BT20 cells than MCF10A cells. **(e) ***HJURP *protein levels are down-regulated by shRNA in MDAMB231 breast cancer cell lines. Part **(f) **shows that MDAMB231 breast cancer cells with shRNA against *HJURP *reduce the sensitivity to radiation.

In order to confirm the relationship between *HJURP *mRNA levels and radiation sensitivity, we selected two cell lines, one had high levels of *HJURP *(MDAMB231), the other had a low level of *HJURP *(T47D), and treated them with different doses of x-ray irradiation. Seventy-two hours after radiation, we measured cell growth and apoptosis using high-content image analysis. Our data showed that the response to radiation in breast cancer cell line MDAMB231 (IC50 = 3.5 Gy) was more sensitive than T47D (IC50 = 8.6 Gy) (Figure 7a). Consistent with radiation sensitivity, MDAMB231 cells had ahigher rate of apoptosis than T47D cells (Figure 7b). Similar results were found in additional cell lines BT20 with high levels of *HJURP *and MCF10A with low levels of *HJURP *(Figure 7c, d). Finally we designed small interfering RNA (shRNA) against *HJURP *and generated stable transfectants in a human breast cancer cell line (MDAMB231). The shRNA down-regulated *HJURP *protein levels by 75%, as assessed by Western blotting assays (Figure 7e). Knockdown of the *HJURP *gene reduced the sensitivity to radiation (Figure 7f).

### Co-overexpression of *HJURP *and CENPA in breast cancer

**Figure 8:**
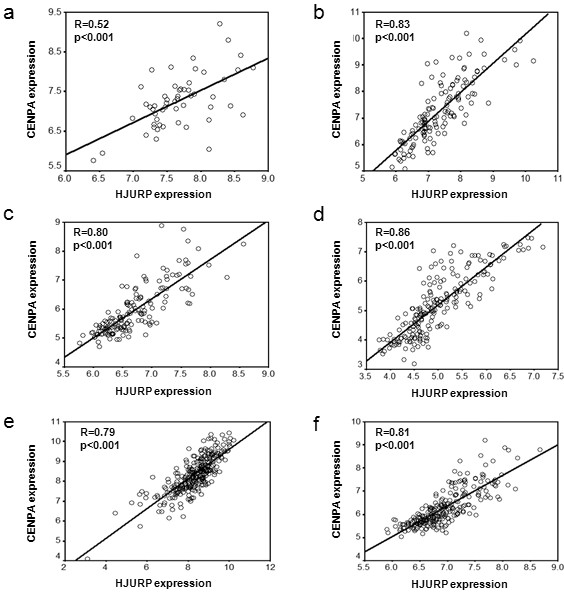
**Correlation between *HJURP *and *CENPA *in mRNA levels**. There is a highly significant and positive correlation between *HJURP *and *CENPA *in mRNA levels within human breast cancer cell lines **(a)**, Primary breast tumors **(b)**, Dataset 1 **(c)**, Dataset2 **(d)**, Dataset4 **(e)**, and Dataset5 **(f)**. R shown is Spearman's rho correlation coefficient.

Recently it has been shown that *HJURP *interacts with CENPA for localization to centromeres and for accurate chromosome segregation. Thus we examined the expression pattern between *HJURP *and CENPA at the mRNA level. Surprisingly, *HJURP *levels were significantly and positively correlated with CENPA levels in human breast cancer cell lines (Figure 8a) and primary breast tumors (Figure 8b). Such highly significant correlation was confirmed in four independent cohorts with breast tumors (Figure 8c, d, e, f).

## Discussion

The current study is the first to report that *HJURP *is overexpressed in breast cancer cell lines and primary human breast cancer compared to non-malignant human mammary epithelial cells and normal breast tissues. High *HJURP *mRNA expression is significantly associated with both shorter disease-free and overall survival which were validated in five independent clinical datasets for breast cancer. Furthermore, *HJURP *is a predictive marker for sensitivity of radiotherapy, indicating levels of *HJURP *mRNA and protein in breast cancer patients are clinically relevant.

Although we found *HJURP *mRNA levels were not associated with ERBB2 status, the mRNA levels of *HJURP *was still found significantly higher in triple-negative (ER negative, PR negative, ERBB2/HER2/neu not overexpressed) breast cancer, possibly due to the fact that a higher *HJURP *mRNA level is significantly associated with ER or PR negative status. Triple negative breast cancer has distinct clinical and pathological features, and also has relatively poor prognosis and aggressive behavior [18-20], consistent with our finding that high *HJURP *expression is associated with a bad prognosis. Furthermore, our studies showed that the prognostic effect of *HJURP *mRNA level on survival is independent of the clinical factors, such as age, lymph node, pathological stage, SBR grade, ER, PR, tumor size, and the molecular subtypes. In addition, we found there is a significant correlation between *HJURP *expression and Ki67 proliferation indices; however, *HJURP *expression is a better biomarker than Ki67 proliferation indices for the predication of prognosis.

It is very interesting to find that the *HJURP *mRNA level is a predictive marker for radiotherapy sensitivity. Our results showed that patients with low mRNA levels of *HJURP *already had a good prognosis and could not get further benefit from radiotherapy, suggesting these patients may not necessarily benefit from receiving radiotherapy. However, patients with high *HJURP *mRNA levels could increase their survival with radiotherapy, but they still had a worse prognosis than those with low levels as found in Dataset 3 (Figure 4c) and Dataset 4 (Figure 5a) where almost all patients received radiotherapy with or without additional benefit. Thus a high level of *HJURP *is overall associated with poor prognosis. Although we note our findings will require replication in additional independent and larger cohorts, our *in vitro *studies further confirmed that breast cancer cells with high levels of *HJURP *are more sensitive to radiation treatment, and even more convincingly, knock down of *HJURP *by shRNA reduces the sensitivity to radiation. The radiation induced more apoptosis in these cells, consistent with clinical findings. A previous report showed that *HJURP *interacts with proteins hMSH5 and NBS1, suggesting *HJURP *is involved in the DNA double-strand break repair process [7]. The understanding of the roles that *HJURP *plays in DNA repair and cell death in response to DNA damage may provide new insights into the molecular mechanisms of breast tumor development and may help to improve breast cancer therapies. In addition, we found that cells with *HJURP *shRNA grew slowly (data not shown), which is consistent with the finding that the double time of cell lines was negatively correlated with *HJURP *protein level, indicating *HJURP *plays an important role in cell proliferation. Thus one of the reasons why the ability of *HJURP *to act as a marker for prognosis and response to radiotherapy may be linked to its control of cell proliferation.

*HJURP *has recently been reported to interact with CENP-A for the purpose of localizing CENP-A and loading new CENP-A nucleosomes on the centromere [11,12]. CENP-A is the key determinant of centromere formation and kinetochore assembly, which regulate the complex job of attaching chromosomes to the mitotic spindle; ensuring that those attachments are correct; signalling a delay in mitotic progression if they are not, and regulating the movements of the chromosomes towards the spindle poles in anaphase. Thus overexpression of *HJURP *in human breast cancer may be similar to overexpression of mitotic kinases, such as Aurora kinases, which induce genomic instability that is one of the hallmarks for tumor development. In this study we showed that *HJURP *mRNA levels are highly significantly correlated with CENPA mRNA levels in human breast cancer cell lines and primary breast tumors. Such correlation is also found in other types of human cancer, such as cancers from lung, ovary, prostate (data not shown), suggesting that compatible mRNA levels of *HJURP *and CENPA might be required for tumor progression. Further investigation of the interaction between *HJURP *and CENPA for breast cancer development will be carried out in our future studies.

## Conclusions

The expression level of *HJURP *has an independent prognostic impact for both disease-free and overall survival in breast cancer, and is a predictive biomarker for radiotherapy. Further investigations of the mechanisms of *HJURP *in tumor development and its association with sensitivity to radiotherapy are clearly warranted.

## Abbreviations

CENP-A: centromere protein A; CGH: Comparative Genomic Hybridization; ER: estrogen receptor; ERBB2: v-erb-b2 erythroblastic leukemia viral oncogene homolog 2: neuro/glioblastoma derived oncogene homolog; GEO: Gene Expression Omnibus; HJURP: Holliday Junction Recognition Protein; PR: progesterone receptor; SBR grading: Scarff-Bloom-Richardson grading

## Competing interests

The authors declare that they have no competing interests.

## Authors' contributions

ZH and GH contributed equally. ZH, GH, SG, MP and NB performed in vitro studies. JHM and GH performed statistical analysis. AS and MEL provided microarray expression and survival data. JHM, EAB, ZH and JWG designed the study, and drafted and revised the paper. All authors read, commented, and approved the final manuscript.
